# Review of research progress in immobilization and chemical modification of microbial enzymes and their application

**DOI:** 10.1186/s12934-025-02791-0

**Published:** 2025-07-18

**Authors:** Walaa A. Abdel Wahab

**Affiliations:** Chemistry of Natural and Microbial Products Department, National Research Centre, Dokki, Cairo Egypt

**Keywords:** Enzymes, Immobilization, Chemical modification, Stabilization

## Abstract

Enzyme stabilization is one of the most critical steps help in applying the enzymes on industrial scale efficiently. Enzymes have use in a variety of areas, including medical, industry, food, and even beauty and cosmetics. The industrial application of enzymes is constantly limited by stability and cost. Enzyme synthesis on a large scale involves multiple steps, therefore stability and repeatability are critical. These two goals are crucial on an industrial scale because they translate to reduced time, effort, and cost. Enzyme stabilization provides the stability and reusability required for successful application. Immobilization with appropriate carriers and conjugation with chemically modified polysaccharides are the most common and low-cost strategies used for enzyme stabilization. These tactics enhance the enzyme’s physicochemical characteristics, making it better suited for industrial applications that benefit our daily lives. This review is an attempt to provide a spot on each method, their progress, benefits and draw backs, and their application.

## Background

Proteins called enzymes function as catalysts to quicken processes by reducing their activation energy. Enzymes, to put it simply, are biological catalysts that accelerate chemical reactions without changing their equilibrium. The ability of an enzyme to withstand denaturation resulting from exposure to various deterrents such as severe pHs, solvents, salts, heat stress, etc., is known as enzyme stability.

In both fundamental and applied enzymology, enzyme stabilization is regarded as one of the most important topics because it provides the means to comprehend the mechanism by which enzymes lose their biological function, which in turn enables the determination of the links between the structural stability of enzyme molecules. The primary objective of applied enzymology is to produce stable, usable enzymes. These enzymes typically exhibit modest activity and may break down quickly through a variety of mechanisms [1]. Thus, it plays a crucial role in determining whether an enzyme is available for commercial usage.

The process of denaturation involves applying an external stressor or compound, such as a strong acid or base, a concentrated inorganic salt, or an organic solvent, to proteins or nucleic acids of the enzyme, causing them to lose the quaternary, tertiary, and secondary structures that are present in their native state. For instance, altering the pH will have an impact on the charges on amino acid molecules, preventing amino acids from being drawn to one another. As a result, the enzyme’s active site and overall shape will alter.

Enzymes play a pivotal role across a wide range of chemical industries, including food processing, energy production, pharmaceuticals, and the development of biopesticides. They offer an environmentally friendly alternative to traditional chemical catalysts, contributing to greener and more sustainable industrial processes. For instance, microbial biopesticides—derived from microorganisms that produce bioactive compounds such as secondary metabolites and growth-inhibitory enzymes—are increasingly recognized as eco-friendly substitutes for synthetic pesticides. This growing interest is largely driven by concerns regarding the environmental persistence, toxicity, and potential food contamination associated with conventional agrochemicals [2].

To ensure enzymes function effectively under industrial conditions, enhancing their stability is essential. The method of stabilization, along with the choice of support material, should be tailored to the specific application. Several strategies have been explored to improve enzyme stability, including immobilization, protein engineering, chemical modification, and the use of soluble additives [3]. Among these, immobilization is widely employed due to its ability to improve enzyme reusability, resistance to harsh conditions, and ease of separation from reaction mixtures. Economic considerations also play a crucial role in selecting support materials. Natural polymers such as chitin and its derivative chitosan have gained attention as cost-effective, biocompatible, and biodegradable supports. Chitosan, in particular, possesses multiple functional groups that facilitate covalent or ionic enzyme attachment, making it an attractive candidate for various biotechnological applications [4].

We are going to focus on the two most popular methods, they are easier and cheaper in preparation and application than other techniques, in this review: immobilization (solid support) and chemical modification (soluble support).

### Immobilization

Immobilization often refers to a delay in mobility. By binding to a solid substrate, enzymes become immobile, which limits their mobility and increases their stability and usability [5]. Immobilization is a primary technique for stabilizing free enzymes [6]. Since the 1960s, it has been the focus of much research [1], and it is today widely used in numerous biotechnological applications as well as scientific studies.

Immobilization’s primary objective is to increase an enzyme’s resistance to temperature changes, solvents, pH changes, pollutants, and impurities.

**Fig. 1:**
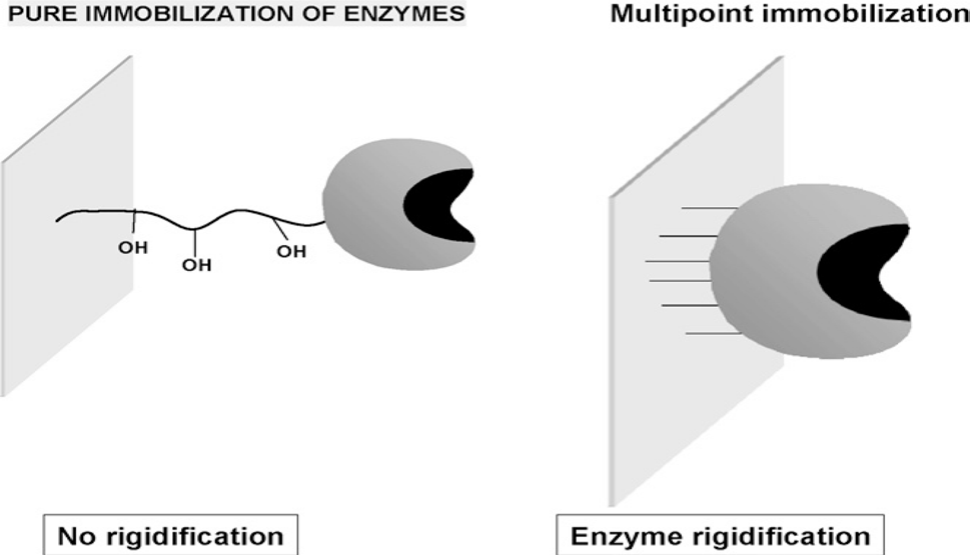
Rigidity effect of immobilization on enzyme stability

Additionally, it makes it simple to separate the enzyme from the reaction mixture (substrates and products), and it provides stiffness (Fig. 1) and multiple reusability, all of which significantly reduce the cost of enzymatic products. The enzyme’s immobilized form facilitates a dependable and effective reaction technology and streamlines its applications [7].

Important factors that affect the immobilization process’s efficiency and, in turn, the cost of production are the support material and immobilization technique employed [8].

### Techniques for immobilization

**Fig. 2:**
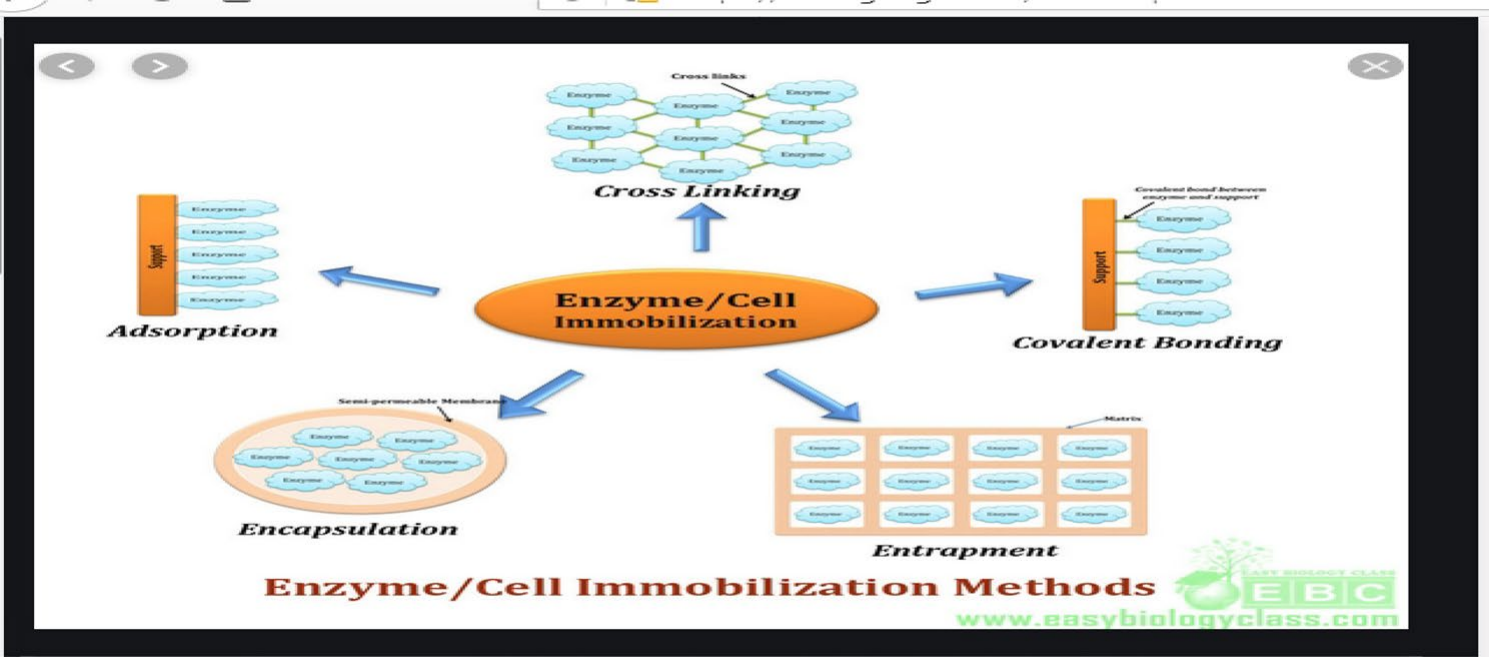
Enzyme immobilization methods

As shown in Fig. 2, there are five basic techniques for immobilizing enzymes: adsorption, covalent binding, crosslinking, encapsulation, and entrapment. It should be noted that no technique is ideal for all molecules or applications, and that no technique is even perfect when using the same enzyme from different sources because each enzyme differs in its structure, properties, active site and its ponds. The physico-chemical characteristics of the target enzyme and the supporting material, in general, dictate the selection of an efficient immobilization technique [7].

### First: adsorption technique

**Fig. 3:**
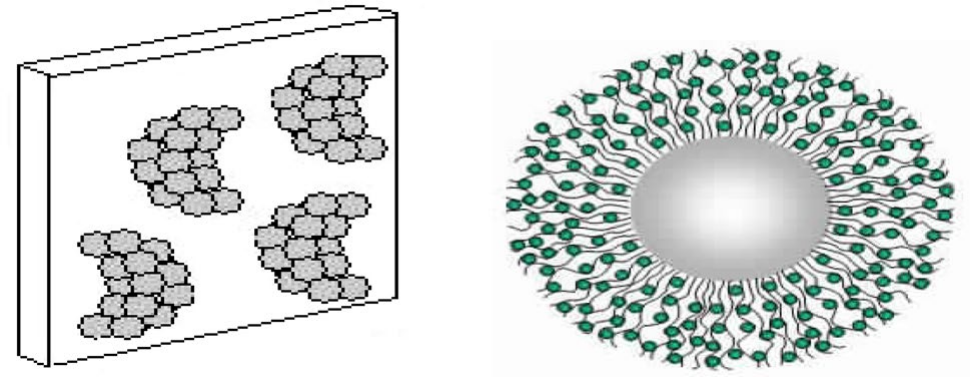
Immobilization by adsorption

It is the most traditional and basic technique for immobilization as it is the easier and doesn’t include strong and deep ponds formation between the enzyme and the support (Fig. 3). The method just entails combining the enzyme with an adsorbent support material under the proper pH and ionic strength conditions for a certain amount of time. After that, the immobilized material, the solid support, is collected and thoroughly cleaned to get rid of any residuals of the free enzyme. Weak forces, such as salt linkage, hydrogen bonds, ionic bonds, hydrophobic bonds, and van der Waals forces, combine the enzyme with the matrix surface [9].

**Fig. 4:**
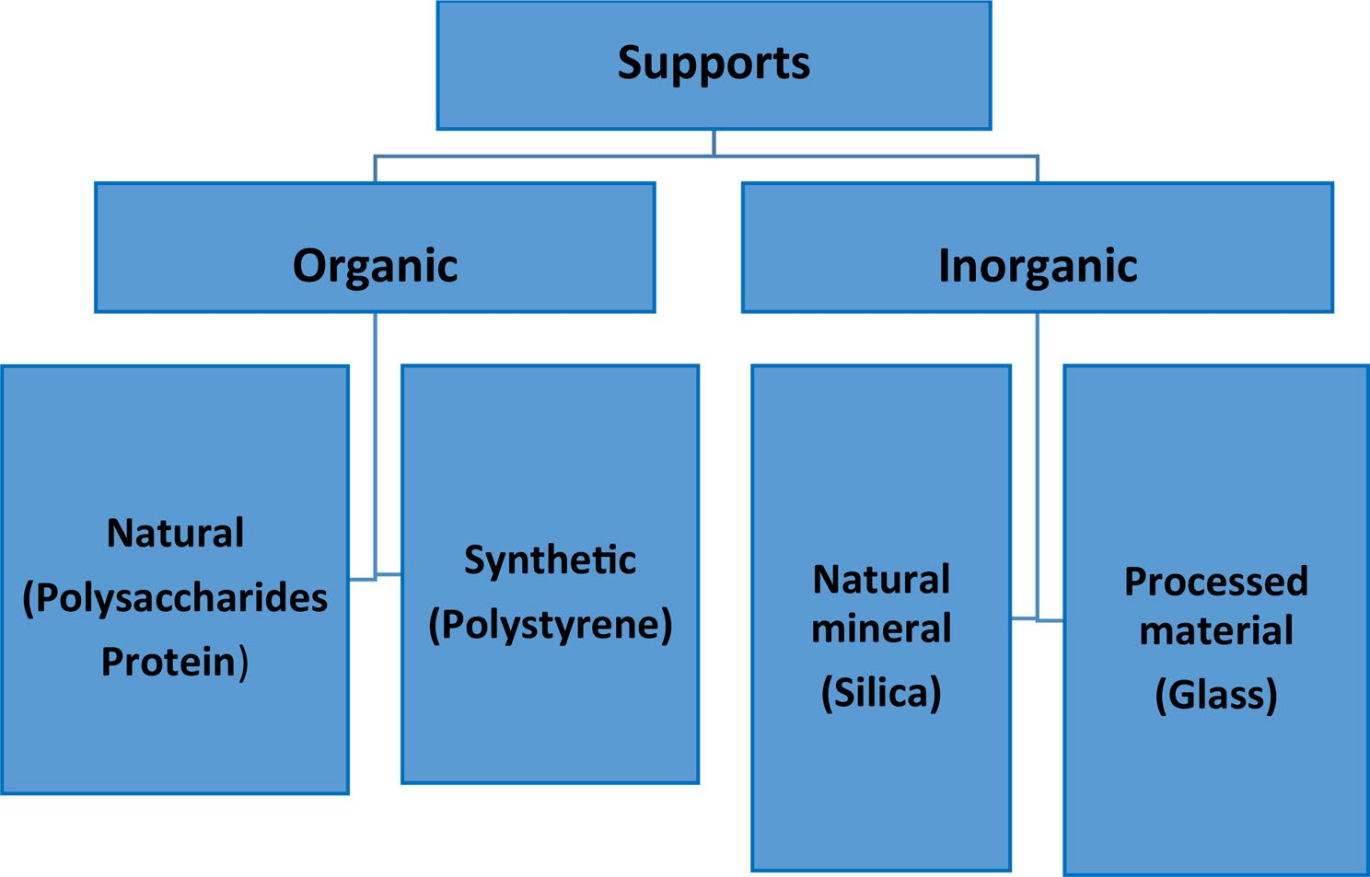
Classification of supports used in adsorption

Supports used for adsorption-based immobilization can generally be divided into two categories based on their origin: organic and inorganic (Fig. 4). The three most widely used inorganic carriers are titania, hydroxyapatite, and silicas. In terms of the organic ones, these comprise manmade chemicals, primarily polymers, and natural compounds such as chitin, chitosan, alginate, and cellulose [10].

Eco-friendly carriers with high cation exchange and good water-holding capacity, such as coconut fibers, have been widely used in adsorption techniques; irreversible binding capacity of microcrystalline cellulose, high enzyme retention capacity of kaolin through chemical acetylation, and large surface area of micro/meso-porous materials with thiol functionalization are ideal supports for this method because they are well suited for oxidation-reduction reactions [11].

Additionally, eco-friendly materials of biological origin reduce production costs and avert the emergence of moral dilemmas. Because of its effectiveness and long-term robustness, supports composed of biocompatible meso-porous silica nanoparticles (MSNs) have recently been exploited for bio-catalysis in energy applications [12].

### Benefits of the adsorption process include

- Reversibility, which permits the carrier to be reused in addition to enabling the purification of enzymes.
- Easy, fast, and sheepish approach.
- A high level of activity retention due to the lack of chemical change.

### Adsorption method drawbacks

- Straightforward enzyme leakage caused by desorption forces such as high pHs and ionic strengths brought on by weak ponds like salt linkage, hydrogen bonds, ionic bonds.
- Restriction imposed by the assistance.
- Product contamination involving the enzyme.

As a result, many experiments such as covalent attachment and cross-linking have been created recently to address this inherent disadvantage [8]. But because of these delays mentioned previously, enzymes immobilized by adsorption are protected against contact with hydrophobic crossing sites, clumping, and proteolysis [13].

### Second: covalent bonding technique

**Fig. 5:**
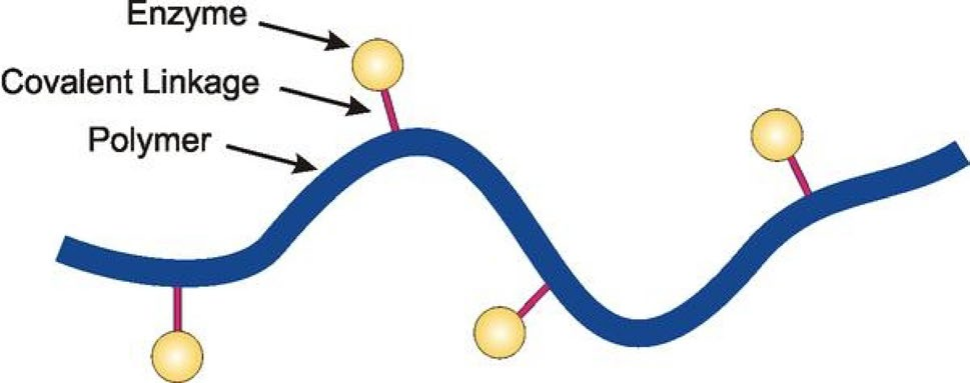
Immobilization by Covalent bonding

It is one of the most often used techniques Fig. 5 for creating stable complexes by covalent binding between the functional groups of enzyme molecules and the carrier matrix. It is important to note that, in order to prevent the enzyme from becoming inactive during immobilization, the functional group of the enzyme that will be covalently linked with the support matrix must not be necessary for enzymatic activity. Typically, this entails binding through the carboxylic group, as in aspartic and glutamic acids, the amino group, as in the side chains of lysine, and the thiol group, as in cysteine [14]. When a reaction process does not require an enzyme in the final product, covalent immobilization is typically used; this is the criterion for selecting this technology [9].

Generally speaking, covalent binding involves the following two steps: Utilizing linker molecules like glutaraldehyde or carbodiimide, the carrier surface is first activated. The process by which the enzyme and activated carrier are covalently coupled. According to Brena and Batista [15], the activation step produces an electrophilic group on the carrier material, which enables the carrier to couple with the powerful nucleophiles on the enzyme.

**Fig. 6:**
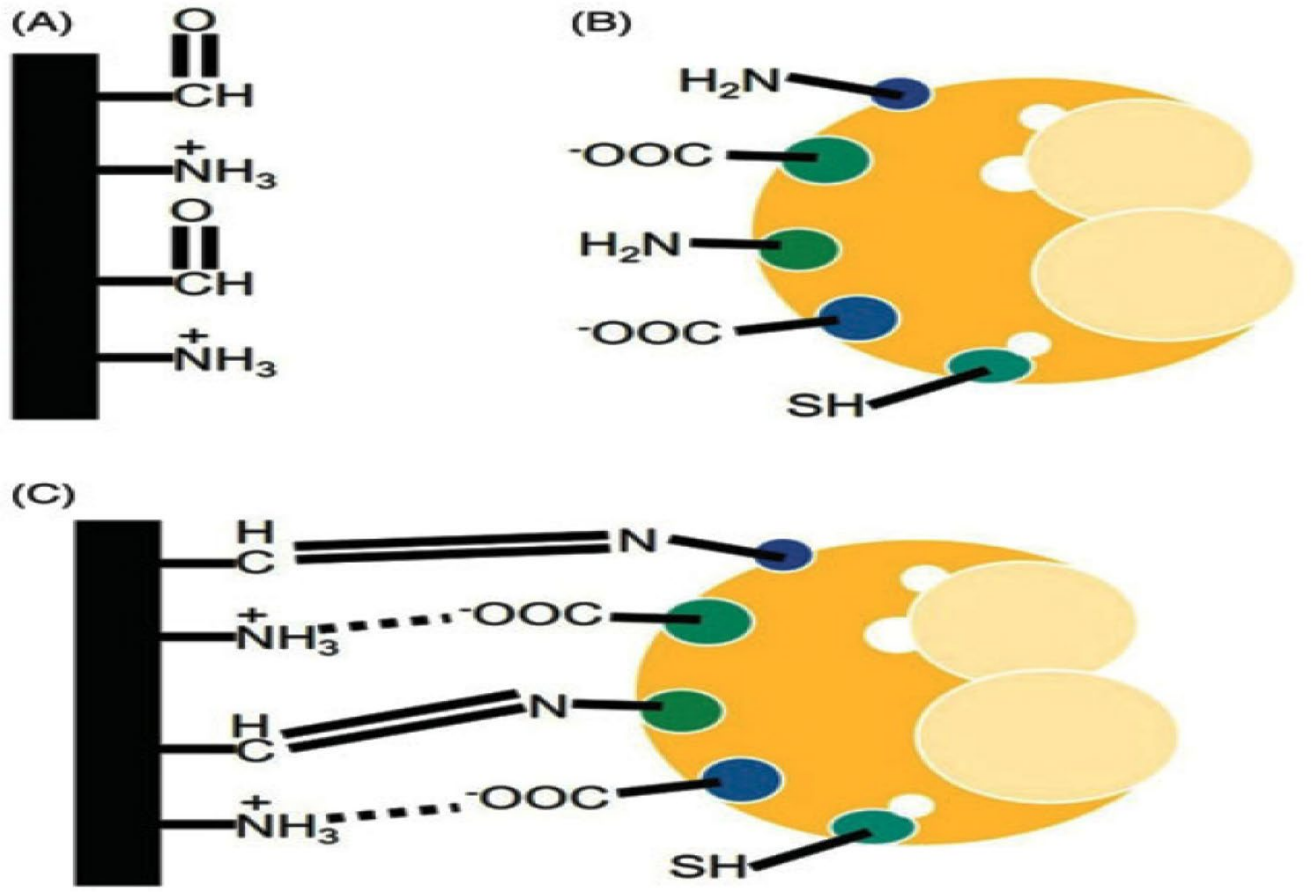
Schematic representation of multipoint enzyme immobilization: (**A**) Support material with functional groups, (**B**) enzyme with functional groups, and (**C**) multipoint cross-linked enzyme immobilization [13]

Glutaraldehyde or carbodiimide, two multifunctional reagents, are utilized as linker molecules in the activation stage. They form covalent bonds with the enzyme to act as a bridge between the carrier and the enzyme. The manner of action of these activation chemicals varies: the carbodiimide tends to bind to pre-activated support and then establishes a covalent link with the enzyme, whereas the glutaraldehyde group occupies the immobilization surface to form the so-called self-assembled monolayer (SAM). As a result, the linkers vary depending on the substance utilized. The carrier material can be an inorganic, natural, or synthetic polymer; membranes such as polyacrylamide; porous glass; agarose; or porous silica. Additionally, there are various immobilization techniques (applying a thin membrane attached to the transducer or directly onto the transducer surface). It is important to note that multipoint covalent bonding, which couples the enzyme molecule with the functionalized carrier through various amino acid residues, can also result in stabilization using this technique (Fig. 6).

### Benefits of immobilization via covalent bonds

- 1- Due to the covalent bond’s strength and stability, there is no enzyme leakage.
  2- Because the enzyme is localized on the carrier, there is easy contact with the substrate.
  3- Better control over the amount of immobilized enzyme and high homogeneity of the produced SAM layer.
  4- Improved thermal stability of the immobilized form compared to the free one [14].

### Covalent bond immobilization has the following drawbacks

- 1- Relatively expensive due to the high cost of suitable supports (e.g., Agaroses and Eupergit C). These days, researchers are focused on producing high-quality, inexpensive carriers to address this lag.
  2- The potential loss of enzyme activity resulting from denaturation, which occurs when enzymes undergo chemical modifications to acquire a functional group (e.g., the active site of the enzyme’s involvement in bond formation, leading to an orientation mismatch in the enzyme; [8].
  3- Relatively longer incubation period compared to adsorption due to the many hours required for the SAM to develop and the enzymes to subsequently bind to it.
  4- Chemical purity and more intricate processes need to be taken into account.

### Third: the cross-linking method

In this kind of immobilization, the enzyme molecules are joined to form a huge, complicated structure in three dimensions. Chemical or physical techniques can be used to accomplish this [16]. Typically, chemical techniques entail employing bi- or multifunctional reagents (such as glutaraldehyde, toluene diisocyanate, or dicarboxylic acid) to build a covalent bond between the enzymes. Physical bond cross-linking has made extensive use of flocculating agents (polyamines, polyethylene eimine, polystyrene sulfonates, etc.) and many phosphates. Since the weak mechanical qualities of the aggregates reflect severe constraints, this technique is rarely utilized alone; instead, it is frequently employed to strengthen other immobilization techniques.

- **Benefits of the crosslinking process include**:

- 1- Support-free immobilization.
  2- Increasing the enzyme’s stability by making the structure stiffer as a result of the enzyme molecules aggregating.

- **The crosslinking technique has several drawbacks, including**:

- 1- The inapplicability of the method for many enzymes due to the harshness of the chemicals utilized.
  2- If the cross-linking reagent interacted through the active site, there may be a possible partial loss of enzyme activity or complete inactivity [8].

### Fourth: encapsulation technique

The process of encapsulation entails encasing the enzymes in various types of semipermeable membranes. Since the enzyme is free in solution but constrained in space, it is similar to entrapment. Although the semipermeable membrane allows tiny substrates and products to flow freely through, enzymes are unable to enter or exit the capsule structure. Numerous materials, such as nylon and cellulose nitrate, with diameters ranging from 10 to 100 μm, have been used to create microcapsules. Drugs, enzymes, and cells have all been successfully encapsulated via ionotropic gelation of alginates [17]. Conversely, silica-based nano-porous gel glasses have been used at the nanoscale level to stabilize some proteins by encapsulating them. Menaa et al. [18] investigated the encapsulation and stability of certain proteins using nano-porous sol-gel glasses based on silica.

### Benefits of the encapsulation approach include

- 1- Enclosing the enzymes within the cell.
- 2- The ability to co-immobilize cells and/or enzymes in any desired configuration to suit a particular application.

### The encapsulation technology has the following drawback

If the products build up quickly, diffusion issues could cause the membrane to break [8].

### Fifth: entrapment technique

Using this method, enzymes are contained within a permeable membrane made of synthetic, natural, or polymeric networks, allowing substrate-product contact while keeping the enzyme within the network [9].

In contrast to adsorption and covalent binding, this method restricts the mobility of the enzyme molecules by means of the gel’s framework structure. Controlling the porosity of the gel is important to avoid cell or enzyme leakage while allowing free passage of substrate and product. Because it shields the immobilized enzyme from microbial contamination by dangerous cells, proteins, and enzymes in the microenvironment, the carrier in this approach also serves as an impediment and can be beneficial [19].

**Fig. 7:**
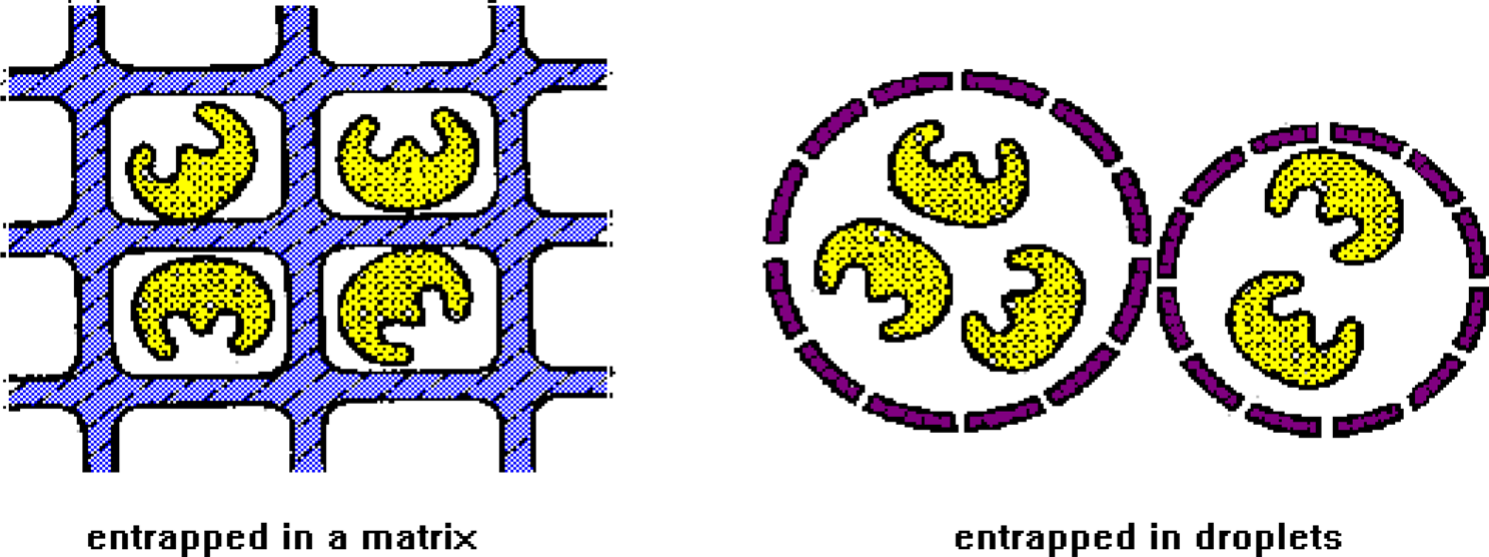
Immobilization by entrapment

Ionotropic gelations, as described in Fig. 7, is a process that combines the enzyme with a polyionic polymer material such as carrageenan, or it involves crosslinking the polymer with multivalent cations such as hexamethylene diamine in an ion-exchange reaction to form a framework structure that deceives the enzyme.

### Benefits of the entrapment method include

- 1- High enzyme loading.
- 2- Quick, inexpensive, and mild conditions are required.

### The entrapment approach has certain drawbacks

- 1- The carrier-induced release of enzymes.
  2- Diffusion limitations: the product’s escape from the enzyme and the substrate’s transfer to the enzyme [8].

### Previous trials in making new carriers

Numerous studies sought to immobilize enzymes through the use of various carriers with various origins and characteristics. They were all focused on carriers with novel, affordable, highly stable, and reusable characteristics. A carrier is generally regarded as being ideal when it has multiple locations for enzyme attachment, excellent physical and chemical stability, good biocompatibility, and is inexpensive [20]. For instance, the process of making glass from raw materials has significant implications for science, business, and technology. Glass made from fly ash, metallurgical slag, and basalt as a raw material is less expensive than glass made from oxide powders of elementary technical grade [21]. In 2019, Ahmed et al. [22] synthesized a new support from basalt as the raw material in immobilizing caseinase using covalent binding and physical adsorption techniques. According to this study, after seven consecutive cycles, the carrier using the covalent bond approach maintained 57.5% of its initial activity and displayed a higher thermodynamics value. α-amylase was immobilized by >Abdel-Hameed et al. in 2022 [23] on a novel carrier, sugilite (a collection of glass compositions derived from modified basalt rock), based on the sugilite formula.

Amidated pectin-polyethylene imine-glutaraldehyde (AP-PEI-GA) immobilizer for xylanase was introduced by Saleh et al. in 2023 [24]. It was able to hydrolyze various agro-industrial wastes into reducing sugar and xylooligosaccharides while maintaining 100% activity for 20 consecutive cycles. It is important to note that this carrier functions as a covalent immobilizer and was first made from pectin, an anionic biopolymer that may be derived from a variety of plant sources, including citrus fruits, apples, mangoes, and passion fruits. In order to immobilize inulinase, Wahba et al. [25],, developed a novel carrier using gellan gum, alginate, and soy protein. The enzyme can potentially be used again for twenty cycles thanks to the immobilization method. Additionally, it extended the enzyme’s shelf life to two months at 4 °C. After seven hours, the immobilized inulinase was able to hydrolyze 61.8% of the 2% inulin into fructose. Awad et al. [26] developed a novel protease immobilization carrier based on the high density of surface amino groups present in hyperbranched polyamidoamine. This carrier was constructed by surface-modifying κ-carrageenan gel beads, which improved the stability of the enzyme and allowed it to retain almost 89% of its initial activity after 8 weeks of storage at 4 °C. Additionally, the immobilized protease was able to effectively remove silver from used X-ray film for six consecutive cycles. It is important to note that Abdel Wahab and Ahmed [27] were able to extract silver by employing free protease.

### Nanotechnology-driven materials

Recent advancements in nanotechnology have significantly enhanced the performance and stability of microbial enzymes through the development of nanostructured supports and materials. Nanomaterials such as graphene, metal-organic frameworks (MOFs), carbon nanotubes, and magnetic nanoparticles offer a high surface-to-volume ratio, unique surface chemistry, and superior mechanical and thermal stability, making them ideal for enzyme immobilization. Graphene-based platforms, in particular, have demonstrated remarkable potential in biosensing due to their excellent conductivity, biocompatibility, and functionalization capacity, enabling high sensitivity and selectivity in biological applications [28].

Furthermore, rational design of nanobiocatalysts, incorporating enzymes with nanoscale carriers, has opened new avenues in food technology and environmental applications by improving enzyme activity, reusability, and operational stability under industrial conditions [29].

In the context of renewable energy, nanomaterial-immobilized enzymes have shown substantial promise in biofuel production, where they enhance the catalytic conversion of biomass-derived substrates due to improved mass transfer and protection of the enzyme structure under harsh reaction environments [30]. These innovations underscore the pivotal role of nanotechnology in overcoming key limitations of native enzymes and expanding their practical applications across biotechnological domains.

### Novel approaches to enzyme immobilization

New generations of immobilized enzymes have been developed using novel approaches in response to the pressing need for stable and reusable enzymes in the field of industrial biotechnology. Some of these novel approaches will be covered in this review.

### Immobile and nano-porous gold

Immobilization on nano-porous gold, or NPG, has become one of the newest carriers throughout the last ten years. This support is easy to produce, offers a high surface to volume ratio, a variety of hole sizes, and is compatible with multiple enzyme immobilization methods. The NPG support is especially intriguing for the immobilization of redox enzymes used in biosensor and biofuel cell applications because it makes it possible to create stable, large-surface-area electrodes. Though immobilizing glucose oxidase on NPG is believed to be a more sophisticated type of glucose sensors, immobilizing laccase and associated enzymes on NPG is an example of biofuel cells [31]. Yan et al. [32] immobilized xylanase using a dealloyed nanoporous gold metallic sponge structure with variable porosity and high biocompatibility, maintaining around 80% of the activity of the free enzyme.

Moreover, there are potential uses for NPG in medicine delivery. Its high surface area-to-volume ratio, adaptability to organic compounds, including medications, simplicity of shape and size modification, and biocompatibility are what give it its promises. Also, NPG nanostructures have the ability to produce the photothermal effect. This action can be employed to either eliminate cancer cells locally by heating them, or to release medications with therapeutic relevance under control [33].

### Microwave irradiation technique

This method is used to immobilize enzymes in meso-cellular siliceous foams (MCFs). It was applied to the immobilization of penicillin acylase (PA) and papain. The findings indicated a notable reduction in the process time and an improvement in the enzyme loading where the highest loading of papain was found to score 1.26 times higher than when no microwave help was used. In contrast, the activities of immobilized penicillin acylase and papain using the microwave-assisted methodology scored 1.39 and 1.86 times higher than those using the traditional method. The results of enzyme adsorption in MCFs were obviously improved by microwave irradiation [34].

### Photo-immobilization technology

It was discovered that when UV light at 365 nm is applied to horseradish peroxidase (HRP) or glucose oxidase (GOD) with a photoreactive polymer, the reactive nitrene immobilizes the enzyme molecules through covalent bonding in 10 to 20 min [7]. This approach has the potential to be significant in the immobilization of biomolecules, irrespective of their functional groups, due to the nitrene nature of insertion into the C-H bond.

(HRP) and (GOD) have been immobilized onto the photoreactive cellulose membrane by sunlight [35]. They stated that 21,625 lx was the ideal level of immobility when employing sunshine. It is important to note that exposure to sunlight, as opposed to 365 nm UV radiation, produced better immobilization outcomes than in dark or on untreated surface. This method works on both large and small scales and doesn’t require any special equipment.

### Enzyme mediating immobilization

It is a new method used to manufacture solid protein formulations, avoiding immobilization challenges and reducing enzyme activity loss due to denaturation. Wong et al. [36] selected enhanced green fluorescence protein (EGFP) and glutathione S-transferase (GST) as model proteins using a neutral Gln-donor substrate peptide.

### Carrier-free enzyme immobilization

The necessity to get beyond several of the drawbacks of the several common immobilization methods, such as the immobilized enzyme’s activity being diluted due to the usage of additional polymers as a carrier, gave rise to this technology. Additionally, challenging and time-consuming studies are required for the significant loss of native enzyme activity, especially at large enzyme loadings, and the development of the immobilized enzyme. Researchers have created carrier-free techniques, such as cross-linked enzyme aggregates (CLEAs) and crystals (CLECs), in response to the aforementioned problems [37].

Because no additional support carrier is used, the carrier-free immobilization technique offers highly concentrated enzyme activity, increased catalytic stability, and cheap manufacturing costs [13].

#### (CLECs) and (CLEAs)

**Fig. 8:**
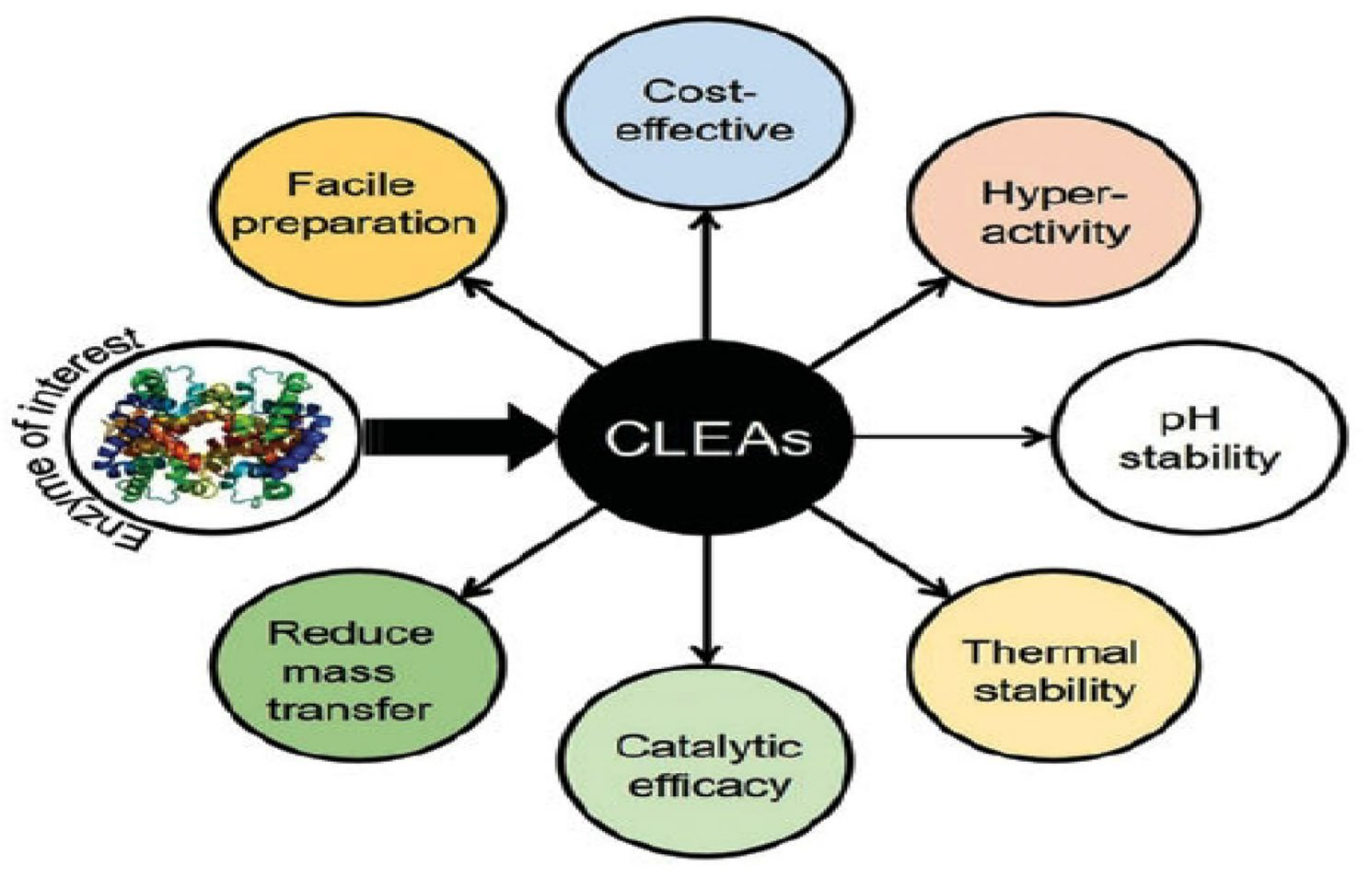
Potential advantages of CLEAs

The creation of Water-insoluble particles, CLEAs, and CLECs has recently demonstrated an intriguing effect in improving the enzyme’s operating stability towards heat, pointing to a viable carrier-free immobilization method. Furthermore, according to Yang et al. [38], both CLECs and CLEAs give their biocatalysts the qualities of being more stable, reusable, and physically stronger than their native forms. CLEAs use unpurified enzymes and are based on the aggregation of an enzyme prior to its chemical cross-linking. Conversely, CLECs entail the crystallization of pure enzymes, followed by chemical crosslinking using any appropriate crosslinker. Figure 8 illustrates a number of CLEA benefits, such as their rigidity, ease of usage, and ability to employ unpurified enzymes [39].

The primary disadvantage of this approach is that, despite CLECs’ notable activity and mechanical stabilities in organic solvents, their production requires the purified enzyme. Because they are made from crude enzyme, CLEAs are environmentally favorable and eco-friendly bio-catalysts for industrial applications. Furthermore, the possibility to effectively co-immobilize two or more enzymes in CLEAs provides the capacity to catalyze several reactions. It is important to note that only one enzyme can be incorporated into a particle during the creation of CLECs [40].

CLEAs technology retains significant activity and protein loss in extreme circumstances such as high temperature and surfactant (SDS). For example, tyrosinase-based CLEAs (produced by enzyme precipitation with ammonium sulphate followed by glutaraldehyde cross-linker) retained 62 and 75% of their original activity after incubation in pure acetone and 1,4-dioxane, respectively. In contrast, free tyrosinase demonstrated insignificant or no residual activity at high solvent concentrations (40% v/v) [41].

### Protein-coated microcrystals (PCMC)

In addition to the conventional freeze-drying process and enzyme powders, protein-coated microcrystals (PCMC) serve as a promising one-step approach that has demonstrated substantial potential to increase the catalytic activity of hydrolases (i.e., lipases and proteases) in low-water conditions. Usually, PCMC is created by quickly dehydrating water-soluble micro-sized carrier crystals and allowing enzymes to adhere to their surface [13]. The creation of combi-PCMC is a smart concept for both cascade (a series of consecutive enzyme activation reactions) and non-cascade catalytic bioprocesses, and it helps to combine two distinct hydrolytic enzyme specificities. Moreover, cross-linked PCMC has been seen as a better design than traditional PCMC, with the potential to yield better results [42].

### Application of immobilized enzymes

**Fig. 9:**
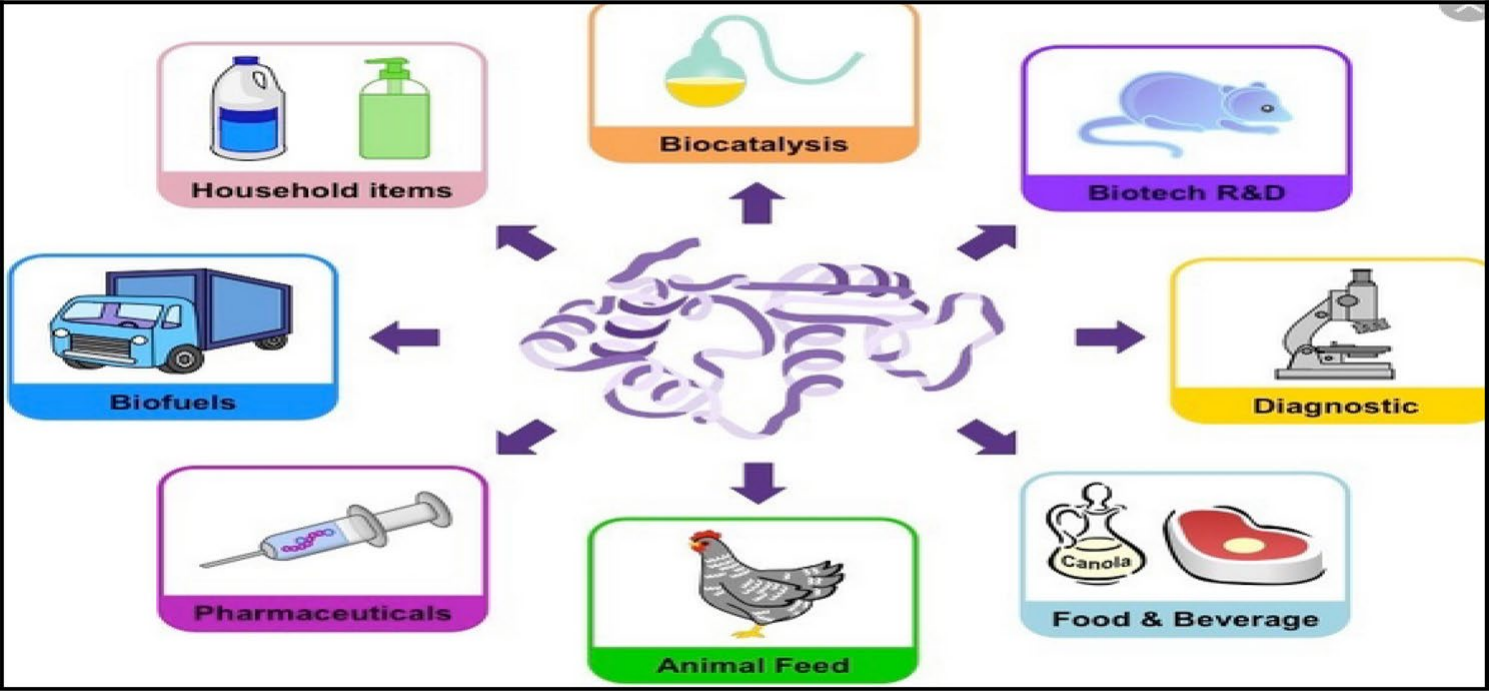
Different industrial applications of enzymes

Enzymes are widely utilized in a wide range of industrial applications, including those in the food, medical, textile, and domestic industries. A few examples of enzymes used in industrial settings are shown in Fig. 9. Enzymes are frequently employed in industry in their immobilized state on inert and insoluble carriers in order to boost their stability and multiple reusability. As we’ve already discussed, the immobilization technique and carrier type affect the characteristics of immobilized enzymes.

**Fig. 10:**
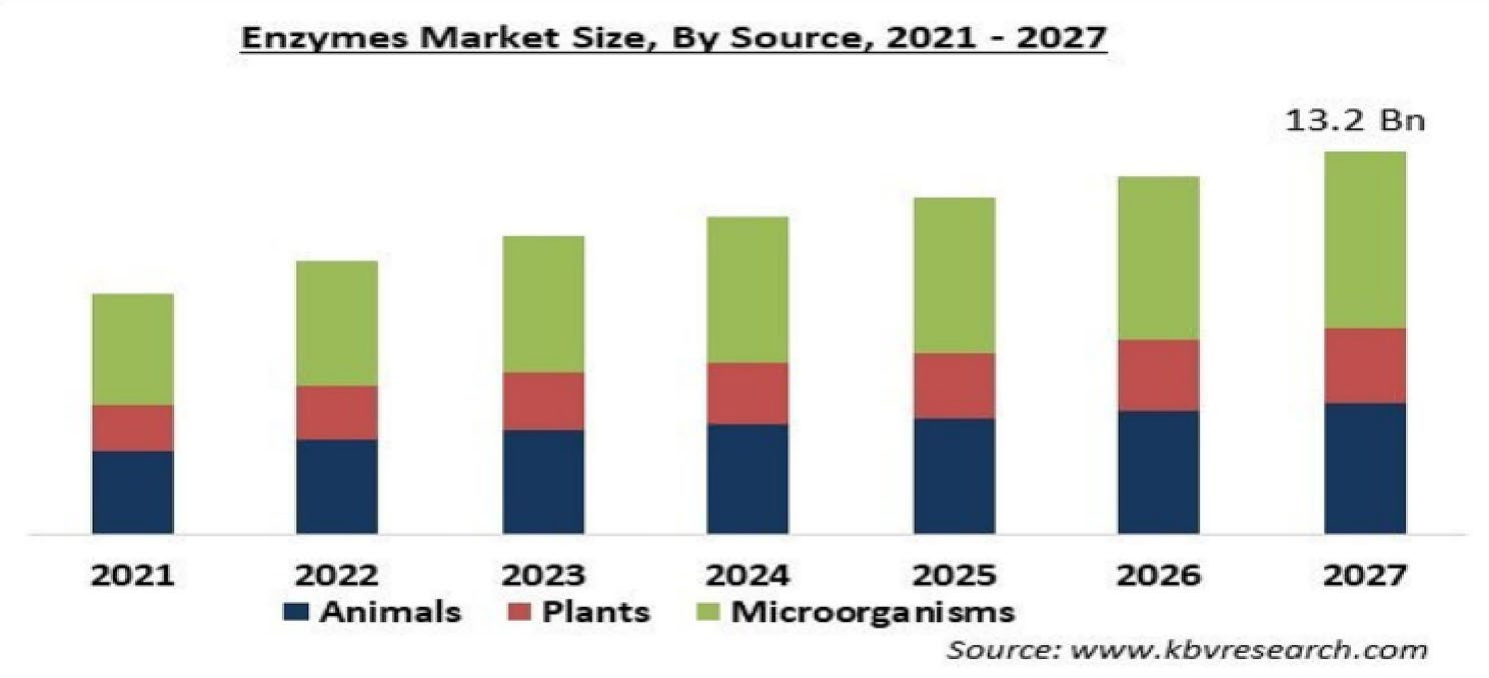
The global enzyme market analysis from 2021 to 2027

The market for enzymes is expanding annually, as shown in the chart below. Figure 10 depicts the global enzyme market analysis from 2021 to 2027.The market for enzymes has expanded quickly in the last several years. By 2024, it is expected to have grown from $11.78 billion in 2023 to $13.52 billion, with a 14.8% compound annual growth rate. The market for enzymes is projected to grow at a 6.8% annual rate between 2023 and 2032. In the sections that follow, we will look at some of these uses.

### In medicine

#### As biosensors

Biosensors offer a wide range of uses, such as the identification of pathogens and toxins in food and water, as well as biomarker detection for medical diagnostics [43]. These are mechanical, chemical, optical, or electrical instruments that can identify and quantify particular chemicals in complicated samples. The optimal biosensor should be able to distinguish between different species based on the immobilized recognition molecules on its surface and react to low analyte concentrations.

Enzymes have been employed as biosensors (Table 1) to quickly and precisely diagnose a wide range of illnesses.

**Table 1:** Some of enzymes acting as biosensors

Analyte	The enzyme used in detection	Reference
Urea	Urease	Yadav et al., [44].
Cholesterol	Cholesterol oxidase	Sekretaryova et al., [45].
Uric acid	Uricase	Arslan [46].
Penicillin	Penicillinase	Yildiz & Toppare [47].
Alcohols	Alcohols oxidase	Liu et al., [48].
Glucose	Glucose oxidase	Updike & Hicks [49].
Amino acids	L-Amino acid oxidase	Lata & Pundir [50].

The great specificity and sensitivity of biosensors, which enable the identification of a variety of analytes in complex samples (blood, serum, urine, or food) with little sample preparation, are what give them their significance [7].

### Production of antibiotics

The use of immobilized enzymes in the manufacturing of antibiotics is recognized due to their environmentally friendly nature, ability to function at room temperature, and ability to avoid the need for organic solvents and chemical procedures. There have been reports of the industrial use of enzyme synthesis for numerous biologically significant antibiotics, including penicillin G acylase (PGA) and β-lactam [51, 52].

#### Cancer therapy

A promising approach to treating cancer is the delivery of enzymes (in the form of immobilized form on nanoparticles, NPs) to target cells in an efficient procedure at the appropriate times and locations together with high bioactivity. According to Sharifi et al. [53], the mechanism of bioactivation involves the healing approach converting prodrug/enzyme molecules into active drug/enzyme molecules upon arrival at the designated spot. By activating enzymes at specific places, the prodrug strategy aims to improve therapeutic potency and mitigate harmful side effects [54].

According to Olesen et al. [55], electroporation therapy, or EPT, is a unique therapeutic approach in which prodrug-actuating enzymes are first introduced into tumorous tissues through various directing strategies. This enzyme directing is then followed by therapy using a safe prodrug that is selectively converted into an anticancer poison by the targeted site’s enzymatic function. It has been established that a number of supports can supply a large number of enzymes needed to activate anticancer prodrugs based on a multi-responsive nanoplatform for precise cancer treatment. Since EPT can transmit prodrug activation, destroy tumorous cells, and initiate it, many studies have indicated that it is a great technique for treating destructive disorders like cancer [56].

Moreover, Asrorov et al. [57] pointed to cytotoxic enzymes and how they are useful weapons in the fight against cancer. A single protein poison molecule should ideally be sufficient to cause the death of a single cell. numerous intelligent delivery methods based on enzyme medications have been developed. A number of these delivery methods have advanced to the point of clinical trials, and a few have even been used in specific cancer therapies. To improve their distribution and targeting, a range of biological, chemical, and physical techniques have been applied to increase their efficiency.

Furthermore, research is constantly being done on the current obstacles and potential uses of NPs in the immobilization of enzymes and the application of these immobilized enzymes to prodrug activation in the cytoplasm of cancer cells.

### Bioremediation

**Fig. 11:**
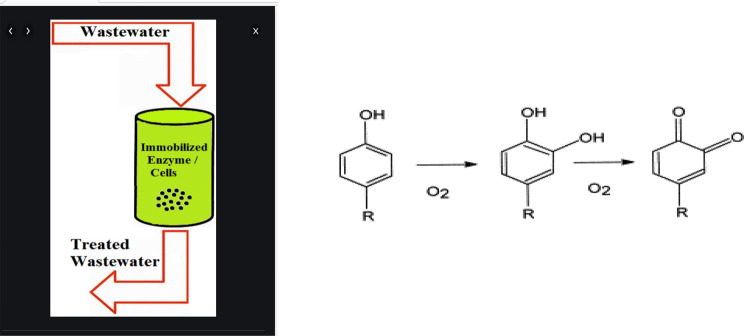
Immobilized Polyphenol oxidase removed phenolic pollutants

One well-known issue is the release of wastewater with a high reactive dye content. Colorless aromatic amines are produced and accumulate as a result, and they have the potential to be extremely harmful and carcinogenic. The elimination of phenolic contaminants from aqueous solutions using the enzymatic technology has generated a lot of interest (Fig. 11). This approach offers an alternative to the conventional chemical and microbiological treatments, which have some significant drawbacks [58].

According to Farag et al. [59], the breakdown of 2, 4- Dichlorophenol and other harmful phenolic and aromatic compounds was improved by immobilizing marine halophilic Bacillus subtilis AAK cultures and its partly purified laccase enzyme by entrapment and adsorption procedures. The reusability of the immobilization technology enables the use of this cost-effective bioremediation strategy.

### The production of biodiesel

One of the most promising applications of immobilized microbial enzymes is in the production of biodiesel, where they serve as biocatalysts for the transesterification of lipids. Compared to chemical catalysts, immobilized lipases offer advantages such as milder reaction conditions, reduced by-product formation, and easier product separation. Moreover, enzyme immobilization facilitates their repeated use, reducing operational costs in large-scale biodiesel production.

**Fig. 12:**
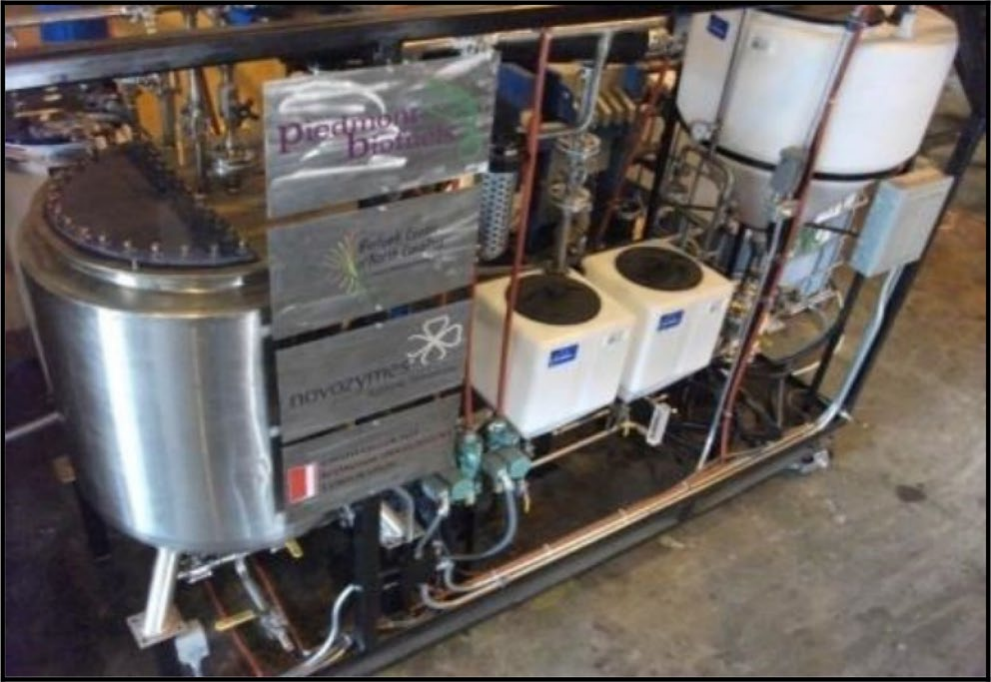
Enzymatic biodiesel processing unit

Because biodiesel may replace fossil fuels, which are not renewable energy sources, it has gained relevance recently. Furthermore, the use of biodiesel, which has been shown to be an environmentally beneficial product, is encouraged by the environmental problems associated with gas emissions from the burning of fossil fuels. By recycling the enzyme, immobilized enzymes are used in the synthesis of biodiesel (Fig. 12) to lower production costs [60].

**Fig. 13:**
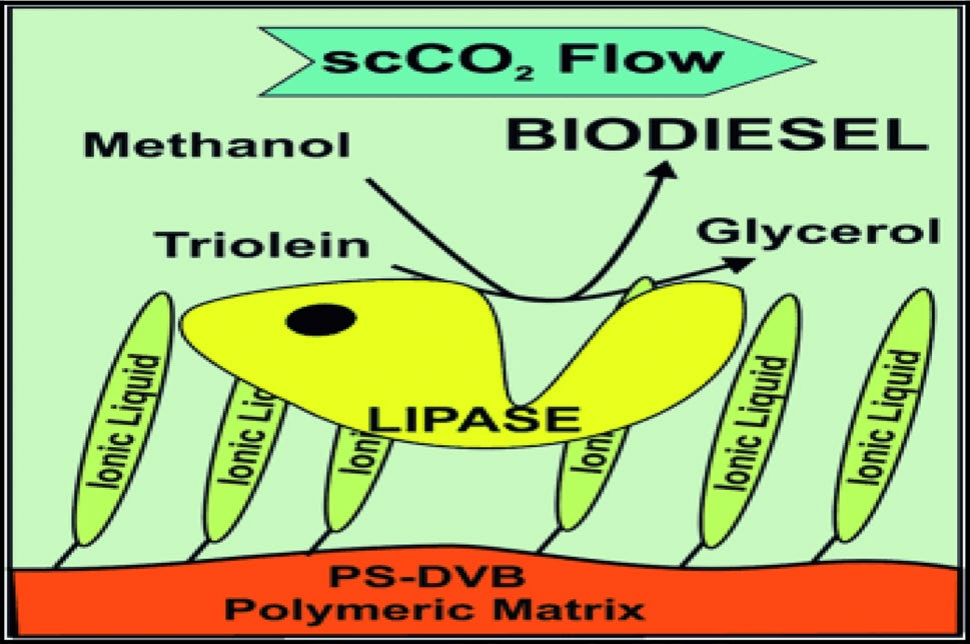
Immobilized lipase for biodiesel production

Recently, most of the researchers have used lipases (Fig. 13) from different sources for biodiesel production (lipase-catalyzed transesterification has been applied for biodiesel production since the glycerol can be removed easily and the purification of fatty acid methyl esters is simple [61].

A recent study by Verma et al. [62] demonstrated the efficient use of bio-nanoparticle-immobilized lipase in the transesterification of algal biomass for sustainable biodiesel production. The authors reported improved catalytic activity and stability, as well as enhanced biodiesel yield, when using bio-nanoparticles as enzyme supports. Similarly, Chandel et al. [63], reported enhanced stability, reusability, and operational efficiency, for Immobilized lipases underscoring the importance of selecting appropriate immobilization methods and support materials to optimize enzyme performance in industrial applications. It is worth mentioning that the integration of nanobiotechnology in enzyme immobilization strategies holds significant promise for developing efficient and sustainable biodiesel production processes. Various nanoscaffold materials—such as nanoparticles, nanofibers, nanotubes, nanopores, nanosheets, and nanocomposites—offered high surface areas for enzyme loading, leading to increased catalytic activity. These nanocarriers not only enhance enzyme stability under operational conditions but also facilitate reusability, which is a critical point in industrial biodiesel synthesis [64].

### Food industry

Immobilized enzymes are very useful in food applications. For example, high fructose syrup can be made by converting glucose to fructose using immobilized glucose isomerase, which results in 42% fructose, 50% glucose, and 8% other sugars [65].

**Fig. 14:**
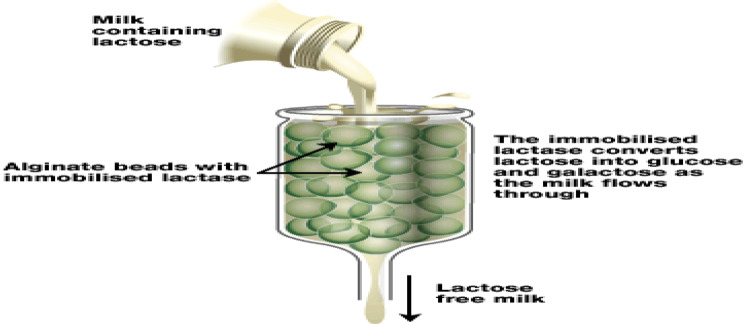
Immobilized lactase for lactose-free milk production for lactose-intolerant people

The lactase enzyme immobilized in alginate beads (Fig. 14) is used to produce lactose-free milk. The corresponding immobilized enzymes are also used for hydrolysis, whey processing, and skimmed milk production. α-amylase immobilized on calcium alginate beads has proven to be a very effective method for starch hydrolysis [66].

Garcia-Quinto et al. [67] noted that the production of nutraceuticals—food additives with positive health effects—has increased the use of immobilized enzymes. Nutraceuticals are isolated using immobilization technology and then added to regular foods to increase their added value. In addition, he pointed out that immobilized food enzymes are superior to the free form in catalytic activity, pH and temperature stability, and operational capacity. Therefore, the possible challenges related to commercial scale-up will be overcome by improvement of novel immobilization techniques to create extremely stable biocatalysts.

### Chemical modification (stabilization through soluble support)

The term “enzyme stabilization by chemical modification” describes the process of attaching an enzyme to polysaccharides that have undergone chemical alteration in order to stabilize the enzyme and improve its physicochemical properties, particularly heat stability.

**The primary benefits** of this technology include the ability to stabilize enzymes quickly and affordably (in comparison to genetic stabilization) by the inclusion of monomeric or polymeric moieties or crosslinking. Additionally, chemical modification makes it possible to introduce a wider variety of chemical groups—groups that determine specificity—to the enzyme structure and structure that are inaccessible through conventional mutagenesis methods. Moreover, it may be done on the appropriately folded enzyme and does not require prior knowledge of the protein structure.

**Fig. 15:**
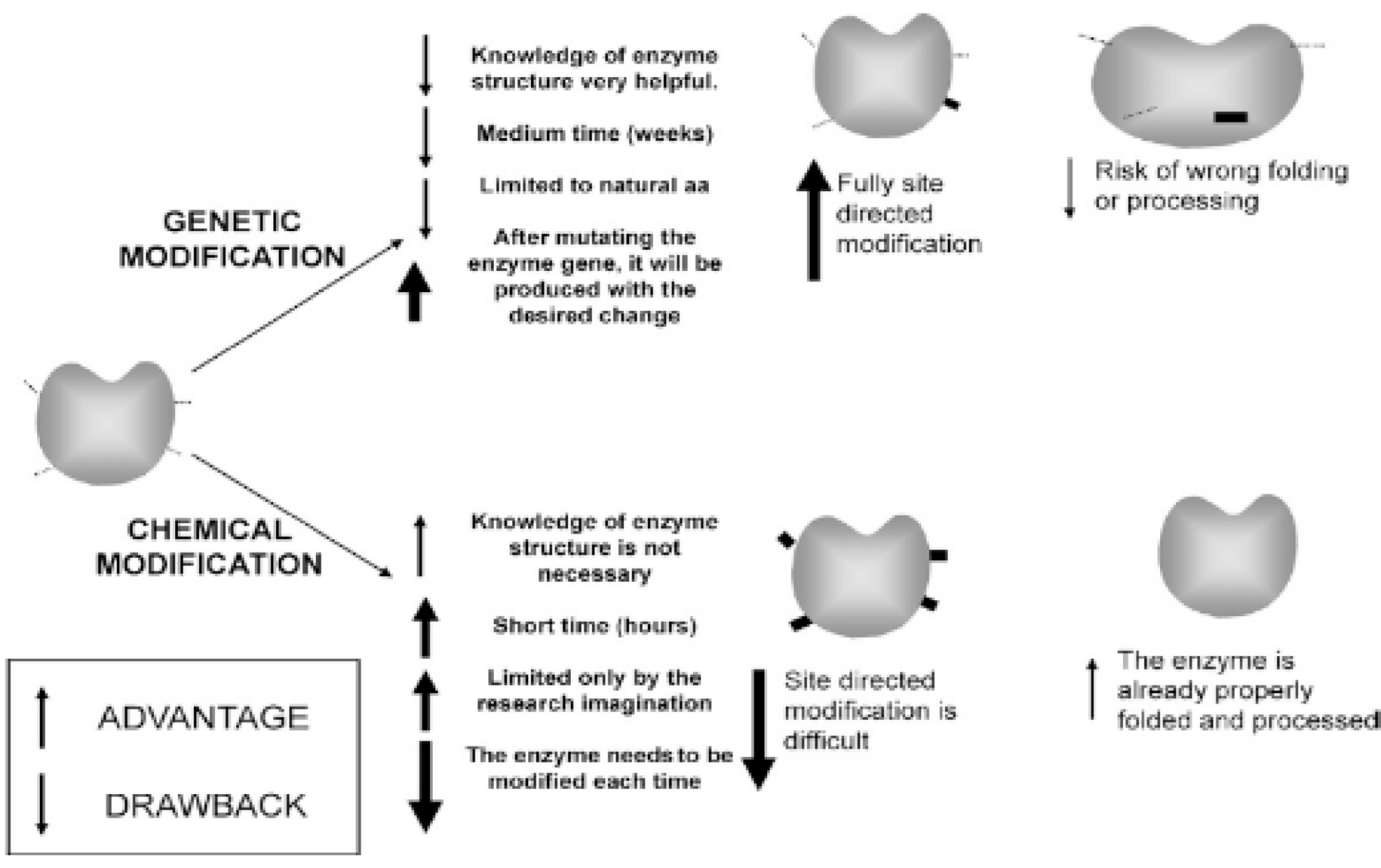
The difference between chemical modification and genetic stabilization

**The main downside** of the chemical modification strategy is a lack of control over the degree and region chemistry of the reaction; however, this disadvantage can be overcome by combining site-directed mutagenesis with chemical modification. This combination results in a fast, regulated, and multifunctional technique that produces well-characterized homogeneous products [68]. The accompanying graphic (Fig. 15) illustrates the comparison between chemical modification and genetic stability.

### Polysaccharides

Polysaccharides are a type of macromolecules with molecular weights (Mw) ranging from tens of thousands to millions. They are typically composed of more than ten monosaccharide units connected by glycosidic linkages [69].Animals, plants, algae, and microbes are among the species that create polysaccharides.

Recently, polysaccharides have involved large attention due to their complex Due to their intricate structure and wide range of biological properties—such as antioxidant, hepatoprotective, antibacterial, immunological, and anticancer properties—polysaccharides have garnered a lot of attention lately. However, certain natural polysaccharides have been shown in numerous trials to exhibit strong anti-tumor properties without any discernible adverse effects [70]. It is important to note that certain polysaccharides have been used to create functional foods that are preventive against cancer and others that work as active molecules or precursors for the treatment or inhibition of cancer [71].

Despite the fact that polysaccharides have a wide range of bioactivities, they are not always sufficient and require improvement. Numerous investigations have demonstrated a strong correlation between their pharmacological or biological actions and their molecular structure. Current studies indicate that molecular modification can alter the dimensional structure, kinds of substituent groups, and Mw of polysaccharides, all of which can affect the bioactivities of the resulting compounds [72].

Numerous chemical modification techniques have been established to date, including; acetylation, phosphorylation, selenium, carboxymethylation, and sulfation. Chemical alteration generally imparts new biological properties and greatly affects polysaccharide biological activity, expanding the applications of polysaccharides [71].

### Methods of chemically modified polysaccharides preparation

Chemically altering a polysaccharide’s structure to produce a polysaccharide derivative with increased or novel biological activity is known as chemical modification of polysaccharides [73]. Its altered functional groups, altered Mws, degradation of polysaccharides, and increased solubility all contribute to the alteration in its biological activity. The most common chemical alteration techniques are acetylation, phosphorylation, sulfation, and carboxymethylation, among others. It is worth mentioning that, polysaccharide-based nanoparticles, such as those made from chitosan and alginate, offer biocompatibility, biodegradability, and functional groups suitable for chemical modifications. These modifications can enhance enzyme binding, stability, and activity [74].

### Conjugation of chemically modified polysaccharide with enzymes

By combining an appropriate weight of the polysaccharide with probing enzyme units and letting them sit at 4 °C for a set amount of time (typically overnight), the activated polysaccharide and enzyme conjugate. Subsequently, 50% acetone was used to precipitate the conjugated enzyme. The efficiency of the conjugation process was then assessed by resolving the precipitate and measuring the amount of recovered enzyme activity. It is important to note that each enzyme has a different ideal incubation period for the conjugation process. The carbohydrate polymers turned out to be important in the stability of enzymes, as we previously discussed. This may be connected to the glycosylated enzyme molecule’s creation of more intra- and intermolecular bridges. Also, they use their conjugated enzymes to convey various biological activities.

Numerous researchers have used the conjugation technique to stabilize enzymes. For instance, *T. longibrachiatum* KT693225 exochitinase demonstrated significant stability when conjugated with activated agar for just two hours, achieving a high percentage of restored activity (79.74%) as reported by Abdel Wahab et al. [75].

It is worth mentioning that, the chemical modification process of polysaccharides results in adding some biological activities like activating the immune system [76], resistance to cancer [77] and antibacterial activity [78] which in turn gains the conjugated enzyme same biological activities.

## Conclusion

Enzymes are the key to many critical reactions in our bodies and communities. They are essential in a variety of industrial applications because they provide fast, low-cost, and environmentally friendly processes. As a result, numerous recent research has focused on enzymes in all aspects. Stability is a key characteristic that encourages enzyme application. Enzymes can be stabilized using a variety of ways. This review is an attempt to focus on two of the most important stabilization techniques: immobilization and conjugation with chemically modified polysaccharides. There is also a brief description of how polysaccharides are prepared for conjugation. These strategies improve the enzyme’s physicochemical properties, making it more appropriate for industrial uses that benefit our daily lives.

## Data Availability

No datasets were generated or analysed during the current study.
